# Redox-active cerium oxide nanoparticles protect human dermal fibroblasts from PQ-induced damage

**DOI:** 10.1016/j.redox.2014.11.007

**Published:** 2014-11-22

**Authors:** Claudia von Montfort, Lirija Alili, Sarah Teuber-Hanselmann, Peter Brenneisen

**Affiliations:** Institute of Biochemistry and Molecular Biology I, Medical Faculty, Heinrich-Heine University, Düsseldorf 40225, Germany

**Keywords:** Cerium oxide, Skin cancer, Fibroblasts, Redox-active nanoparticles, Tumor–stroma interaction

## Abstract

Recently, it has been published that cerium (Ce) oxide nanoparticles (CNP; nanoceria) are able to downregulate tumor invasion in cancer cell lines. Redox-active CNP exhibit both selective pro-oxidative and antioxidative properties, the first being responsible for impairment of tumor growth and invasion. A non-toxic and even protective effect of CNP in human dermal fibroblasts (HDF) has already been observed. However, the effect on important parameters such as cell death, proliferation and redox state of the cells needs further clarification. Here, we present that nanoceria prevent HDF from reactive oxygen species (ROS)-induced cell death and stimulate proliferation due to the antioxidative property of these particles.

## Highlights

• Beneficial, non-cytotoxic effect of cerium oxide nanoparticles on stromal cells.
• Supporting a prospective therapeutical approach of such particles.
• Protection of stromal cells against the oxidative damage.

## Graphical abstract

**Figure:**
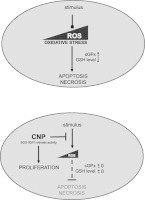

## Introduction

In recent years, nanomedicine has gained a lot of interest. It deals with the therapeutical application/use of particles <100 nm and represents a promising tool in anticancer therapy [1]. Both carrier and non-carrier particles have been used in the last years, with one of the non-carrier particles being cerium (Ce) oxide nanoparticles (CNP). CNP are characterized by a mixed valance state of Ce^3+^ and Ce^4+^; this interchangeable property makes them regenerative [2]. They have been shown to possess both pro-oxidant and antioxidant property, the latter enabling them to scavenge superoxide radicals [3]. CNP exhibit superoxide dismutase (SOD−) and catalase-mimetic activity in a cell-free system [4,5]. In 2011, it was demonstrated that nanoceria reveal an inhibitory effect on the formation of myofibroblasts, an important step in tumor progression [6]. These CNP being non-toxic for stromal cells show a cytotoxic and anti-invasive effect on tumor cells, indicating a bifunctional role of CNP in tumor–stroma interaction [7]. In each therapeutical approach, it should be of uttermost importance that healthy (stromal) cells are not damaged and/or inhibited in their functions, but in reality this is often not the case. Therefore, in this study, the question was addressed whether nanoceria protect stromal cells (with fibroblasts as a model) from inducers of apoptotic or necrotic cell death as well as inhibitors of proliferation.

## Materials and methods

Cell culture media (Dulbecco's modified Eagle's medium (DMEM)) was purchased from Invitrogen (Karlsruhe, Germany) and the defined fetal calf serum (FCS gild) was from PAA Laboratories (Lind, Austria). All chemicals were obtained from Sigma (Taufkirchen, Germany) or Merck Biosciences (Bad Soden, Germany) unless otherwise stated. The protein assay kit (Bio-Rad DC, detergent compatible) was from BioRad Laboratories (München, Germany). The enhanced chemiluminescence system (SuperSignal West Pico/Femto Maximum Sensitivity Substrate) was supplied by Pierce (Bonn, Germany). Monoclonal mouse antibody raised against α-tubulin was supplied by Sigma.

## Cell culture

Human dermal fibroblasts (HDF) were established by outgrowth from foreskin biopsies of healthy human donors with an age of 3–6 years. Cells were used in passages 2–12, corresponding to cumulative population doubling levels of 3–27 [8]. Cells were cultured as described [9].

## Synthesis of cerium oxide nanoparticles

Cerium oxide nanoparticles were synthesized in water and in dextran (molecular weight: 1000 Da) using previously described methods [19]. Briefly, cerium nitrate hexahydrate was dissolved in deionized water and the pH of the solution was maintained between 3.5 and 4.0 for uncoated (water-based) nanoparticles. Stoichiometric amounts of hydrogen peroxide and ammonium hydroxide were added to oxidize the dissolved cerium ions as cerium oxide nanoparticles. The pH of the solution needs to be maintained strictly below 4.0 to avoid precipitation of the nanoparticles. For synthesis of dextran-coated nanoparticles stoichiometric amounts of dextran were at first dissolved in deionized water followed by cerium nitrate hexahydrate. The solution was stirred for 2 h followed by addition of ammonium hydroxide (30% w/w). The pH of the solution was kept below 9.5 to avoid precipitation of cerium hydroxide. The resulting cerium oxide nanoparticles were analyzed using UV–visible spectroscopy for determining the oxidation state of nanoparticles and transmission electron microscopy for particle size.

## Cell viability

The cytotoxic effect of CNP was measured by the MTT (3-(4,5-dimethylthiazol-2-yl)-2,5-diphenyltetrazolium bromide) assay [10]. The activity of mitochondrial dehydrogenase, as indicator of cellular viability, results in formation of a purple formazan dye. Briefly, MTT solution (0.5 mg/ml) was added to the cell cultures treated for various times with the nanoparticles. The cells were incubated for an additional hour. The medium was removed and the cells were lysed in dimethyl sulfoxide. The formazan formation was measured at 570 nm. The results were presented as percentage of mock-treated control which was set at 100%.

## SDS-PAGE and Western blotting

SDS-PAGE was performed according to the standard protocols published elsewhere [11] with minor modifications. Briefly, cells were lysed after incubation in 1% SDS with 1:1000 protease inhibitor cocktail (Sigma; Taufenkirchen, Germany). After sonication, the protein concentration was determined by using a modified Lowry method (Bio-Rad DC). 2× SDS-Page sample buffer (1.5 M Tris–HCl pH 6.8, 6 ml 20% SDS, 30 ml glycerol, 15 ml beta-mercaptoehtanol and 1.8 mg bromophenol blue) was added, and after heating, the samples (10 µg total protein/lane) were applied to 10% (w/v) SDS-polyacrylamide gels. After electroblotting, immunodetection was carried out (1:1000 dilution of primary antibodies, 1:20,000 dilution of secondary antibody). Antigen–antibody complexes were visualized by an enhanced chemiluminescence system. Alpha-tubulin was used as internal control for equal loading.

## Release of lactate dehydrogenase

HDF were grown to subconfluence on tissue culture dishes. After removal of the serum-containing medium, cells were cultured in a serum-free medium for 24 h. Thereafter, cells were collected by centrifugation (1500*g* for 10 min at 4 °C). The supernatant was used to quantify enzyme activity of extracellular LDH with the l-Lactate Assay Kit from Cayman Chemical Company (Ann Arbor, MI, USA) following the manufacturer's protocol.

## Population doubling

HDF were seeded with 15,000 cells/well in a 24-well-plate. After treatment with 150 µM of CNP, cells were incubated at 37 °C. After 24 h, cells were washed with PBS and medium was changed. Every day, the cells of 2 wells were trypsinized and counted. With these values, the population doubling was determined.

## Proliferation assay

DNA synthesis was determined by measuring BrdU incorporation into DNA with the BrdU proliferation assay (Merck, Darmstadt, Germany). Briefly, the cells were incubated with BrdU-label for 12 h. After fixation and denaturation for 30 min the fibroblasts were incubated with primary antibody for 4 h at RT. After washing three times with PBS, they were incubated with secondary antibody and after another washing step substrat solution was added. The extinction was measured at 450 nm.

## DHE staining

Subconfluent HDF were either mock-treated or preincubated for 24 h with 150 µM CNP before incubation with 2.5 mM PQ for either 1 min or 1 h. Thereafter, cells were washed with Hank's Balanced Salt Solution (HBSS), incubated with 2.5 µM Dihydroethidium (DHE) for 1 h and washed again with HBSS. DHE is a dye that forms a red fluorescent product (ethidium) upon reaction with superoxide anions. Fluorescence was analyzed via fluorescence microscopy at 570 nm.

## Measurement of intracellular ROS

Generation of reactive oxygen species (ROS) was determined using 2′,7′-dichlorodihydrofluorescein diacetate (H_2_DCF-DA), a dye that diffuses across the lipid membrane into cells and is subsequently oxidized by intracellular ROS forming the highly fluorescent DCF. Subconfluent HDF were exposed to 150 µM CNP in serum-free DMEM in 24-well plates. Untreated subconfluent cells were used as negative controls. Medium was substituted after 24 h by 100 µM H_2_DCF-DA containing Hanks' Balanced Salt Solution (HBSS). DCF fluorescence was detected at an excitation wavelength of 485 nm and emission wavelength of 520 nm in 15 min intervals in a FLUOstar OPTIMA plate reader (BMG Labtech, Offenburg, Germany).

## GSH determination

Total GSH was determined by the GSSG (GSH disulfide) reductase-DTNB (5,5′-dithiobis (2-nitroben- zoicacid) recycling procedure [12]).

## Statistical analysis

Means were calculated from at least three independent experiments and error bars represent standard error of the mean (s.e.m.). Analysis of statistical significance was done by Student *t* test or ANOVA as appropriate.

## Results and discussion

**Fig. 1:**
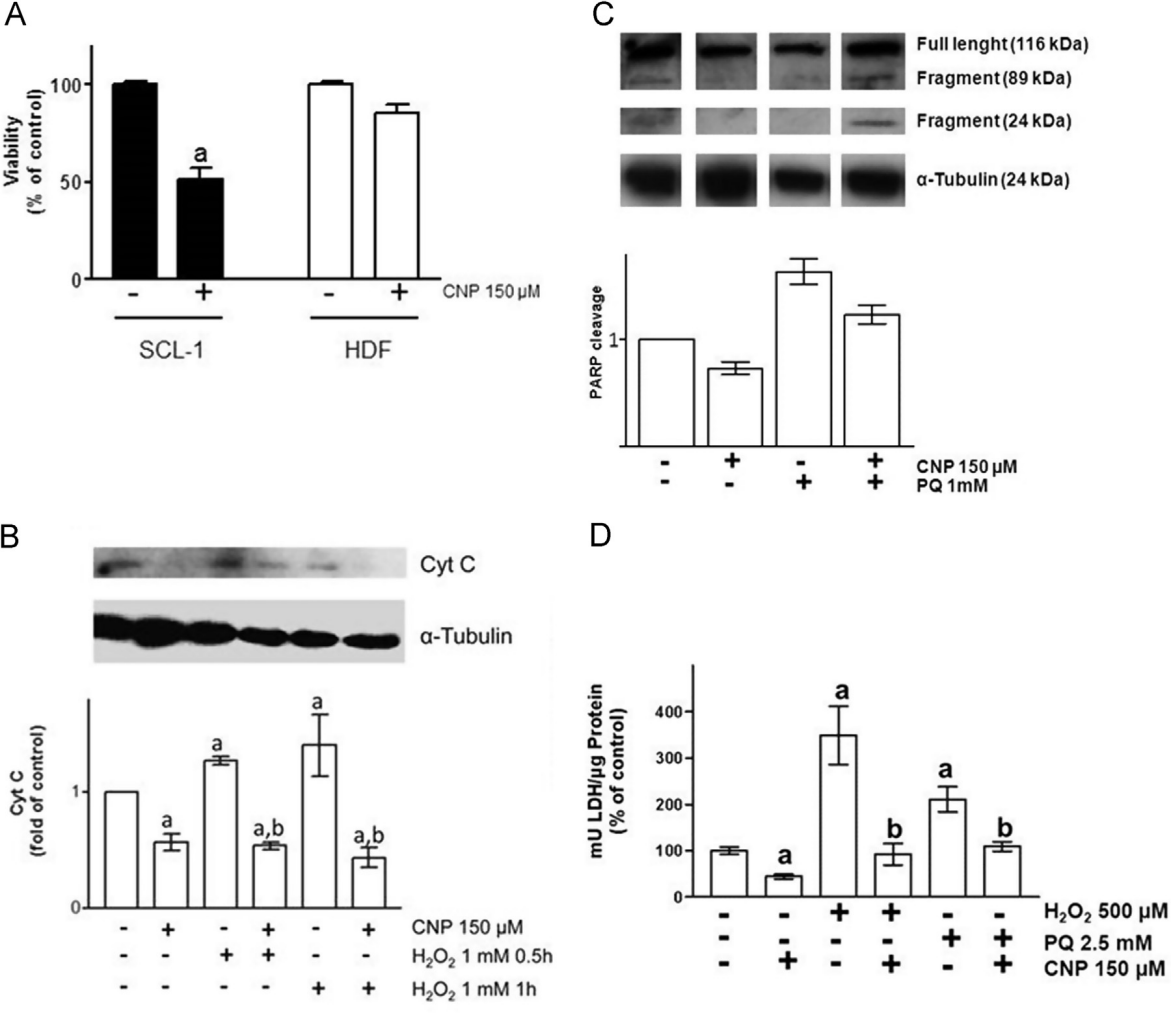
Effect of nanoceria on oxidative stress-induced cell death parameters. (A) Viability of tumor cells (SCL-1) and fibroblasts (HDF) after CNP incubation. (B) Cytochrome c release of HDF after hydrogen peroxide and nanoceria incubation. (C) CNP attenuates PQ-induced PARP cleavage. (D) Nanoceria prevent HDF from oxidative stress induced LDH formation. ANOVA with Dunnet's post-hoc test or Student's *t*-test were used for the determination of statistical significance among treatment groups, as appropriate. Data are means±SEM, *n*=3–6. (a) *p*<0.05 compared to control and (b) *p*<0.05 compared to absence of CNP.

In earlier studies, it has been shown that CNP lower the viability of squamous cell carcinoma cells, but not of noncancerous cells [6]. Herein, these data were confirmed by treatment of the squamous cell carcinoma cell line SCL-1 and human dermal fibroblasts (HDF) with another batch of nanoceria for 48 h CNP (150 µM) lowered the viability of SCL-1 to about 50% compared to the non-treated control, it had no statistically significant effect on fibroblasts (Fig. 1A). To further clarify the effect of nanoceria on noncancerous, healthy cells, the role of these nanoparticles in context of cell death, i.e. apoptosis and necrosis, was investigated. When preincubated for 24 h, nanoceria were able to decrease the apoptotic effects of hydrogen peroxide and paraquat (PQ) (Fig. 1B and C). HDF were incubated with hydrogen peroxide and/or nanoceria. Incubation with 1 mM H_2_O_2_ for either 0.5 or 1 h induces release of cytochrome c from mitochondria to the cytosol, which was attenuated by preincubation with 150 µM of CNP (Fig. 1B). In Fig. 1C it is shown, that the cleavage of PARP induced by PQ is abrogated by CNP.

To not only assess parameters of apoptosis, we also tested for necrotic activity. In this case the occurrence of lactate dehydrogenase (LDH) in the supernatant was measured (Fig. 1D). CNP alone prevented LDH release to 50% of control. Incubation of the dermal fibroblasts with PQ or H_2_O_2_ resulted in an increase of the extracellular LDH level and CNP treatment significantly lowered this LDH release.

**Fig. 2:**
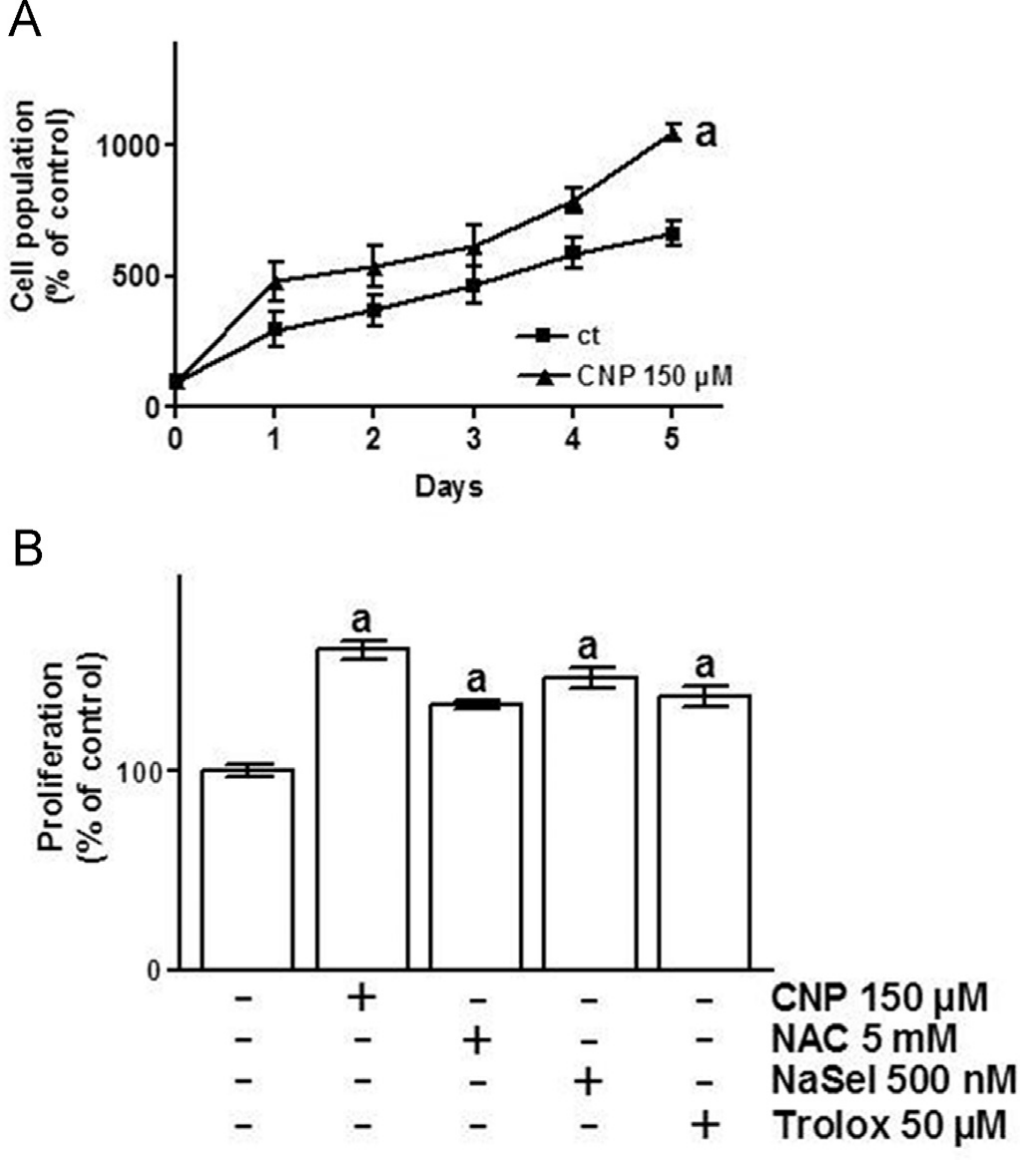
Nanoceria show a proliferative effect on HDF comparable to those of common antioxidants. (A) CNP exhibit a proliferative effect on human dermal fibroblasts (HDF). (B) CNP show the same stimulating effect on HDF proliferation as other prominent antioxidants. ANOVA with Dunnet's post-hoc test or Student's *t*-test were used for the determination of statistical significance among treatment groups, as appropriate. Data are means±SEM, *n*=3–6. (a) *p*<0.05 compared to control and (b) *p*<0.05 compared to absence of CNP.

After investigating the effect of CNP on cell death, the role of nanoceria concerning cell population and proliferation was elucidated. HDF were incubated with 150 µM CNP for up to 5 days, and the population doubling of cells was assessed by cell counting every 24 h after treatment. CNP significantly increased the cell population up to 180% of control after 5 days (Fig. 2A). As the data presented in Fig. 1 hint to an antioxidative effect of CNP, the proliferative effect of these nanoparticles was compared to those of well-known antioxidants (Fig. 2B). BrdU Assays revealed that the applied concentrations of NAC, sodium selenite and Trolox had no cytotoxic effect on the cells. Rather the substances increased proliferation of HDF up to 130–150% of control, with the effect of CNP stronger (about 150%) than the effect of the other compounds.

**Fig. 3:**
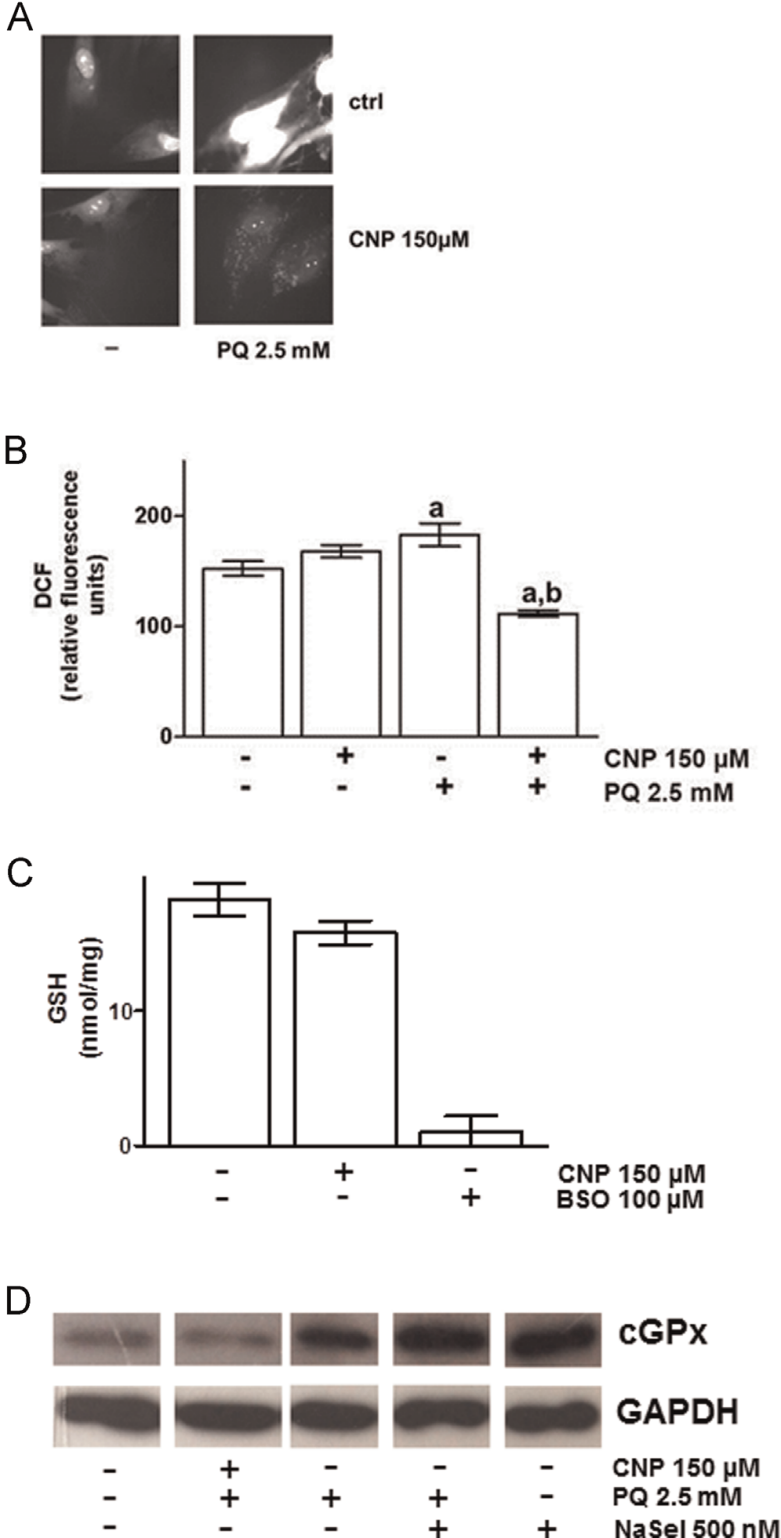
Nanoceria decrease oxidant-induced ROS formation while not affecting GSH or cGPx levels. (A) PQ^2+^-induced superoxide production in HDF is blocked by nanoceria. (B) PQ^2+^-incuded hydrogen peroxide production in HDF is inhibited by nanoceria. (C) CNP did not affect the cellular GSH level. (D) CNP, in contrast to selenite, blunted PQ-induced cGPx expression. ANOVA with Dunnet's post-hoc test or Student's *t*-test were used for the determination of statistical significance among treatment groups, as appropriate. Data are means±SEM, *n*=3–6. (a) *p*<0.05 compared to control and (b) *p*<0.05 compared to absence of CNP.

It is proposed that nanoceria express both pro- and antioxidant activity [7]. To address the hypothesis, that the SOD- and catalase mimetic activity of nanoceria [5,13] is responsible for the here shown effects on cell death and proliferation, we checked the impact of CNP on the intracellular ROS level. It was shown that enhancement of tumor invasion depends on the ROS-mediated generation of myofibroblasts from dermal fibroblasts [14]. First, fluorescence of dihydroethidium (DHE) was monitored. DHE is a fluorescent probe that forms a red fluorescent product upon reaction with superoxide anions. Paraquat (PQ), a well-known O^2·−^ producing redox cycler [15], was used, resulting in an increase of DHE fluorescence (Fig. 3A, upper panel). Preincubation with 150 µM CNP inhibited the PQ-mediated generation of superoxide radicals (Fig. 3A, lower panel). Furthermore, the fluorescent dye dichlorofluorescein (DCF), known to detect peroxides [16], was used for detection of PQ-initiated ROS production. Fig. 3B depicts DCF formation after incubation with PQ with or without CNP preincubation. PQ induces the formation of hydrogen peroxide, but CNP in combination with PQ prevented ROS formation. Finally, to investigate the oxidative state of the cell, the effect of nanoceria on the intracellular GSH level was assessed. CNP did not significantly increase the amount of GSH (Fig. 3C), but prevented PQ induced expression of cellular GSH-peroxidase (cGPx; Fig. 3D), thus indicating an interaction between CNP and PQ and/or PQ induced ROS, that makes a rise in cellular GSH level and an augmentation in cGPx expression unnecessary.

Due to their oxygen vacancies and their ability to switch oxidation states between III (Ce^3+^) and IV (Ce^4+^), resulting in antioxidant activity [4,13], CNP have become a very promising tool in treatment of tumors. Their ability to downregulate tumor growth and invasion without affecting the viability of healthy stromal cells makes them even more suitable for cancer therapy. In this study, we wanted to further investigate the effect of CNP on stromal cells. It was shown here that CNP prevented PQ-mediated PARP cleavage and hydrogen peroxide induced cytochrome c release, indicating a protection from apoptosis. In addition, the necrotic activity induced by various compounds could be abrogated by nanoceria. CNP is not cytotoxic at the indicated concentrations, it rather induced cell growth, which also has been observed by Chigurupati et al. [17]. This increase in proliferation by nanoceria is comparable and even higher than with prominent antioxidants such as NAC, sodium selenite and Trolox, suggesting an effect being due to the antioxidant property of the particles. Additionally, CNP prevented the PQ-dependent formation of superoxide. Superoxide is dismutated to hydrogen peroxide, and it has been shown recently that an increase in hydrogen peroxide due to the activity of SOD mimetics contributes to more damage, when the cellular or a cell organelle's redox system is out of balance and lacks either catalse or GSH to deal with that increase [18]. Here, as seen in Fig. 3B, formation of hydrogen peroxide was also inhibited by nanoceria, which gives another hint for the earlier proposed SOD- and catalse mimetic activity of CNP [5,14]. Interestingly, nanoceria did not increase the cellular GSH level, and also reduced the PQ-induced expression of cellular GPx, an enzyme that uses GSH to detoxify the cell from peroxides by reducing hydrogen peroxide to water while oxidating GSH to GSSG. Here, the reaction of CNP seems to be different than to the antioxidant NaSel. Selenite is a component of the cellular GPx, found in its catalytic center, and so it is not surprising that addition of NaSel *per se* leads to an increase in GPx expression. This increase persists with addition of PQ, indicating an increase of GPx action to defend from PQ induced oxidative stress. With CNP the results are different, CNP lowers the expression of the antioxidant enzyme, indicating a direct interaction of the nanoparticles with PQ induced ROS. This fits with the finding, that CNP per se does not influence the cellular level of GSH (Fig. 3C).

In summary, our results show a beneficial, non-cytotoxic effect of cerium oxide nanoparticles on stromal cells, supporting a prospective therapeutical approach of such particles in context of protection of stromal cells against the oxidative damage playing an important role in pathological disorders such as cancer.

## Conflicts of interest

The authors declare no competing financial interests and conflict of interest.
